# The natural diet composition of young piglets suggests an overlook of fibre and food structure in farmed suckling piglets

**DOI:** 10.1186/s40813-025-00439-4

**Published:** 2025-04-29

**Authors:** Renjie Yao, Hubèrt M. J. van Hees, An Cools, Sebastián A. Ballari, Dominiek Maes, Geert P. J. Janssens

**Affiliations:** 1 Department of Veterinary and Biosciences, Ghent University, Merelbeke, Belgium; 2 Department of Internal Medicine, Reproduction and Population Medicine, Ghent University, Merelbeke, Belgium; 3 Trouw Nutrition, Research & Development, Amersfoort, The Netherlands; 4 Consejo Nacional de Investigaciones Científicas y Técnicas (CONICET) CENAC (Parque Nacional Nahuel Huapi, APN) AR, San Carlos de Bariloche, Rio Negro Argentina

**Keywords:** Wild, Feral piglets, Suckling piglets, Creep feed, Insoluble fibre

## Abstract

**Background:**

The often disappointing intake of creep feed by suckling piglets coincides with a limited ability to cope with challenges such as weaning diarrhoea. Investigating the dietary nutrient profile of piglets (*Sus scrofa*) in the wild may help to improve nutrition for farmed piglets. This study was conducted to analyse the stomach content of feral piglets and their farmed counterparts, and to compare them with the composition of commercial creep feeds. Forty feral piglets (4.6 ± 1.4 kg) living in a wild herd were tracked and legally hunted in the Bahía Samborombón (Buenos Aires, Argentina). Their gastric contents were collected for analysing macronutrients. Twenty-eight farmed suckling piglets of similar ages were sourced from a Dutch research farm, and their stomach contents were collected and pooled into fourteen samples with the same procedure for comparison. Additionally, the composition data of twenty-five commercial feeds was also collected.

**Results:**

A higher dry matter content was observed in the farmed piglets’ stomachs (233 vs. 148 g/kg, *P* < 0.05). The gastric crude protein concentration was similar between both groups but the crude fat concentration was higher in the stomach of farmed animals (525 vs. 238 g/kg DM, *P* < 0.05), while feral piglets consumed more ash and fibre (*P* < 0.05). A similar concentration of non-fibrous carbohydrates (NFC) on metabolizable energy basis was observed by calculation through the NRC’s guideline (*P* > 0.05). Within the fibre content, significantly greater concentrations of neutral detergent fiber (NDF), acid detergent fiber (ADF) and acid detergent lignin (ADL) were observed in the stomach of feral piglets than in those of farmed piglets (282 vs. 36 g/kg, 158 vs. 9 g/kg DM, 53 vs. 3 g/kg DM, respectively, *P* < 0.05). Similar protein concentrations were observed between the gastric content of feral piglets and creep feed, while significantly higher crude ash and crude fat concentrations were found in feral piglets’ consumption (*P* < 0.05).

**Conclusion:**

In conclusion, piglets in natural conditions consume much more fibre—from coarse plant material—compared to farmed piglets receiving creep feed. Although technical performance is distinctly different between nature and farm, it raises the question whether suckling piglets under farming conditions would benefit from a more fibrous and coarser creep feed.

## Introduction

Neonatal nutrition has life-long effects on pigs [5, 16]. Farmed piglets experience a severely shortened suckling period compared to their ancestors. Therefore, they need a robust gastrointestinal tract for this early transition to solid feed and other weaning-associated stressors [29, 33, 48]. To address these challenges, supplemental feed during the lactation period (i.e., creep feed) has been introduced, enabling piglets to become familiar with solid feed already before weaning. However, the current nutrient-dense, highly processed creep feeds often appear unattractive to suckling piglets [45, 50] and have limited effect on gastrointestinal development for weaning preparation or the later life phases [30, 63],Van den [66]. Therefore, diet selection of suckling piglets under free-roaming conditions may inspire to develop novel concepts for creep feed, thereby facilitating a smoother weaning transition.

Wild boar (*Sus scrofa*), native to Eurasia, has become one of the most widely distributed ungulate mammals in the world [42]. As an opportunistic omnivore, wild boar is capable of adapting to diverse food materials, such as plant materials, mud, fungi, and animal matter [4, 70]. The earliest evidence of pig domestication from wild boar dates back to 6600–7500 years BC [1, 13], and the phenotype transformation was promoted through artificial selection [49]. Early selection focused on visible traits, evolving over time to prioritize growth rate, feed efficiency, litter size and stress resistance as genetic understanding advanced [21, 60].

In contrast to wild boar, feral pigs refer to domesticated pigs that were intentionally released or escaped from captivity in recent history. These pigs mostly survived and partly recovered their ancestral morphology and behaviour [27, 69]. However, the feralization cannot be considered as a mere reversal of domestication. During the feralization process, a regained relative brain size and the cell density of the olfactory mucosa has been observed [35, 41], both of which are involved in enhancing food acquisition [12]. A previous study identified distinct traits that partially combine domesticated pigs and wild boars, as well as diet profiles between wild boar and feral pigs living in the same region [54].

In general, studies in diverse wild animal species have reported a drive for particular dietary nutrient profiles that are assumed to match the animal’s requirements [3, 6, 20, 57]. Yet, the long history in promoting genes associated with fast growth and high reproductivity in farmed pigs shifted the pig diet away from their preferences in the wild. Commercial diets largely diverge from the coarse plant matter that feral piglets ingest [68]. During the overturning of soil for underground vegetable items or earthworms [10, 17, 55, 72], pigs living in nature can typically ingest mineral soil when rooting to depths of 15 cm or more. This raises the question if the highly processed, low-fibre diet of suckling pigs in commercial farms is optimal for their health. In some critical periods, e.g. around weaning, piglets are commonly provided with diets in the form of a nutrient-dense, highly digestible and assumedly palatable feed, intending to promote the maturation of their gastrointestinal tract while still mimicking sow milk composition. However, they still appear at risk of developing anaemia or weaning diarrhoea for impaired immune function and increased mortality [7, 26, 39]. As many health problems in pigs are multifactorial, it is not always easy to discern if they are due to nutrition or other factors. For instance, iron deficiency, as the most prevalent, mainly manifests as anaemia in suckling piglets reared indoors. A previous study reported that when pigs participated in an outdoor feeding system and were exposed to soil, benefits of iron supplementation were observed [53]. Meanwhile, post-weaning diarrhoea, closely linked to nutrient levels in the diet, remains a significant issue in pig farms [8, 58]. Identification of the differences between the diet composition of farmed and feral piglets may help to better understand the pathophysiology of nutritional disorders and to improve farmed piglet diets.

Unlike the post-weaning period, the suckling period is focused more on nourishment and ensuring the piglets develop properly rather than solely achieving maximal growth rates, akin to pigs living outdoors where dietary consideration matches maintenance and behavioural needs. Hence, these diets would be a suitable basis for comparison to identify the main differences between the feral and farmed diets independent of performance.

The objective of this study was to identify the nutrient profile of the natural diet of free-ranging feral piglets through stomach content analysis, in comparison with commercial diets and farmed piglets’ stomach contents. This comparison was then used to identify the main nutrient shifts in commercial feed that may need reconsidering.

## Materials and methods

All procedures in the wild were conducted in the legal hunting season and district. As an invasive exotic species, *Sus scrofa* is permitted to be hunted following the provisions of the Rural Code (Law No. 10,081) in the province of Buenos Aires, Argentina. Several hunters were contracted and trained for sample collection before this study. The housing and care of experimental animals applied in the farm was approved by the Dutch Central Animal Welfare Committee under application number AVD2040020184665.

### Sample collection

Forty feral piglets (average body weight: 4.58 ± 1.39 kg), with balanced sex distribution, were traced and collected by hunters in Bahía Samborombón, Argentina from September to November 2018 (austral spring). The feral piglets samples analysed in this study were from the same batch as those described in a previous publication [68]. The protected area is a wildlife refuge, with a large mixohaline wetland. This region experiences cold, wet winters from June to August and hot, humid summers from December to March with prevailing winds from Atlantic Ocean. The average annual temperature is approximately 15 °C (ranging from 9 °C in winter to 21 °C in summer), and the area receives an average annual rainfall of 1000 mm [44]. A great variety of habitats encompasses permanent as well as seasonally flooded freshwater lagoons, marshes, slow-flowing streams, grasslands and islands with trees (primarily *Celtis tala*) and shrubs. Three dominant plant species in the low and middle intertidal saltmarsh were two grasses *Spartina alterniflora* and *Spartina densiflora*, and one amaranth, *Sarcocornia perennis* [32]. The firearms and capture traps were applied with the help of trained dogs according to the previous method of controlling invasive species [43]. Suckling piglets remaining close to feral sows were identified and their age range was estimated by a local hunter (B. Carpinetti, personal observations) based on body size, dentition, and behaviour [65]. Among the piglets tracked in this study, 5, 16, and 19 piglets were approximately 1, 3–6, and 6–16 weeks of age, respectively. The entire content of the stomach of each piglet was collected in an ice box and stored at − 20℃ until later nutrient analyses.

To enable a comparison of the nutrient composition of feral piglets and farmed piglets, the data on stomach content were collected from another experiment conducted at a research farm (Swine Research Centre of Trouw Nutrition, Sint Anthonis, The Netherlands). Two crossbreed piglets (Hypro Libra × Maxter, Hendrix Genetics) around median weight within a litter from each of the fourteen sows (average parity 3.1; ranging from 1 to 6), resulting in a total of 28 piglets, were selected at weaning (d 26), including 13 boars and 15 gilts. The selection was primarily based on body weight, with efforts made to balance sex distribution. The piglets were fed ad libitum with a commonly used commercial creep feed from day 14 of age until weaning. They had free access to water. The feed was formulated for piglets from 14 days of age (pre-weaning) to two weeks after weaning, with its composition presented in Table 1. Three feeding sessions were conducted in the morning sessions, running from 7:30 to 11:20, and afternoon sessions from 14:00 to 17:50. During each session, fresh feed was added to assure that the feed was always available. At weaning, the piglets were euthanized, the entire stomachs containing chyme were weighed, and the stomach contents were stored at − 20 ℃ for later nutrient analysis.

**Table 1:** Composition of creep feed applied in the farmed piglets from 2 weeks prior to weaning (d 26)

Ingredient	Creep feed
Composition, g/kg as-fed basis	
Basal diet^1^	850
Corn starch, heat-treated	150
Total	1000
Calculated nutrients, g/kg as-fed basis	
Moisture	99.8
Crude protein	170
Crude fat	52
Ash	49
Total dietary fibre^2^	120
Soluble dietary fibre^2^	18
Insoluble dietary fibre^2^	102
Lactose	50
Calcium	6.0
Phosphorus	5.8
ME, MJ	13.38
NE, MJ	10.38

ME = metabolic energy; NE = net energy

^1^Basal diet consisted of wheat, barley (58.0%), extruded cereals (7.1%); soybean products (12.0%), including extruded soybean meal (Forcital, Trouw Nutrition, Ghent, Belgium); dairy whey products (6.0%); fats and oils (3.6%); vitamin and minerals (3.2%); sucrose and palatability enhancers (2.5%); wheat protein (2.4%); potato protein (2.4%); synthetic amino acids (2.1%) and organic acids as feed preservatives (0.8%). Vitamins and minerals provided per kilogram total feed: 9,169 IU vitamin A, 2,002 IU vitamin D3, 150 IU vitamin E-acetate, 1.5 mg menadione, 1.6 mg thiamine mononitrate, 4.3 mg riboflavin, 1.9 mg pyridoxine, 30.0 μg cyanocobalamine, 22.4 mg niacin, 12.0 mg calcium D-pantothenate, 683 μg folic acid, 43 μg biotin, 113.7 mg choline chloride, 50.1 mg betaine, 216.8 mg iron, 1.1 mg iodine, 144.2 mg copper, 52.0 mg manganese, 125.6 mg zinc, 0.35 mg selenite

^2^Analyzed content by AOAC 991.43

### Data compilation

We collected the composition data of 25 distinct brands of commercial creep feeds to present the difference between current creep feed formulations and feral piglets ‘ preferences. The inclusion criteria for the creep feed formulations were as follows:

1. already available on the market in Europe;
2. formulated for farmed piglets at or before d 14 of age;
3. feeding them in solid form directly to piglets without mixing them with liquid;
4. availability of nutritional data on at least one macronutrient proportion.

Consequently, the data on crude protein concentration was available for all feeds, crude ash concentration for 11 feeds, and the data on either extract (EE) concentration for 13 feeds. The data on neutral detergent fibre (NDF) was available for only two of them, so the comparison on NDF was abandoned. The data on acid detergent fibre (ADF), hemicellulose, cellulose, and acid detergent lignin (ADL) were not available for these commercial feeds.

### Laboratory analysis

The samples of feral piglets were pooled into 13 samples based on age ranges, and due to the similar ages of farmed animals, the samples were pooled into 14 samples based on body weight. The samples were milled, weighed, and placed in a preheated oven at 103℃. Four hours later, the container was removed from the oven, and placed in vacuum desiccators for 30 to 45 min to cool down. They were weighed and the dry matter (DM) was calculated.

The crude protein, fat, fibre and ash in the stomach content of feral piglets and farmed piglets were analysed using proximate analysis [71]. The Kjeldahl method was applied to determine the amount of protein (N × 6.25) in the samples. To determine the fat concentration, fat was extracted from the samples using diethyl ether after acid hydrolysis. The extract was washed and dried, in order to determine the crude fat content [18].

To determine the ash, the sample was combusted at a temperature of 550 °C after which the residue was weighed [18]. The analysis of cellulose, hemicellulose and lignin was based on previous methods described in the literature [22].

The concentration of non-fibrous carbohydrates (NFC) was calculated to estimate the enzymatically digestible or relatively fermentable carbohydrates, such as mono- and oligosaccharides, starches, and some pectins and beta-glucans [15]. Non-fibrous carbohydrates (NFC) were calculated with the following formula:

$${\text{NFC}}\left( {g/kg,\;{\text{DM}}} \right)\; = \;1000{-}\left( {{\text{Crude}}\;{\text{Protein}} + {\text{Ash}} + {\text{Crudefat}} + {\text{NDF}}} \right)\left( {g/kg,\;{\text{DM}}} \right)$$

The predicted metabolizable energy (ME, kJ/g) of macronutrients in the stomach contents was calculated through the factors based on NRC’s guidelines [52]: 22.0 kJ/g protein, 34.0 kJ/g fat and 17 kJ/g NFC. The proportions of CP, EE, and NFC were approximately calculated relative to the total energy provided by these three macronutrients and represented in a ternary plot based on ME.

### Statistical analysis

A general linear model was applied to evaluate the difference between gastric content in feral piglets and farmed piglets. The age of piglets at dissection was included as covariate. The body weight was regarded as covariate on dry matter comparison. The Tukey–Kramer correction was applied for post-hoc multiple comparisons. The linear regression model in SPSS 28.0 was applied for computing the determination coefficient between the consumption of NDF and ADF, and the difference in slopes and intercepts was compared by an analysis of covariance (ANCOVA). The mean values plus the standard deviation of results were presented, and the comparison between gastric contents of feral piglets and commercial diets was conducted via an unpaired, two-sided T-test. Differences were considered significant when the *P-*value < 0.05. Statistical analyses were performed using SPSS version 28.0 (IBM SPSS Inc., USA).

## Results

### The nutrient composition of gastric contents of feral and farmed piglets

**Fig. 1:**
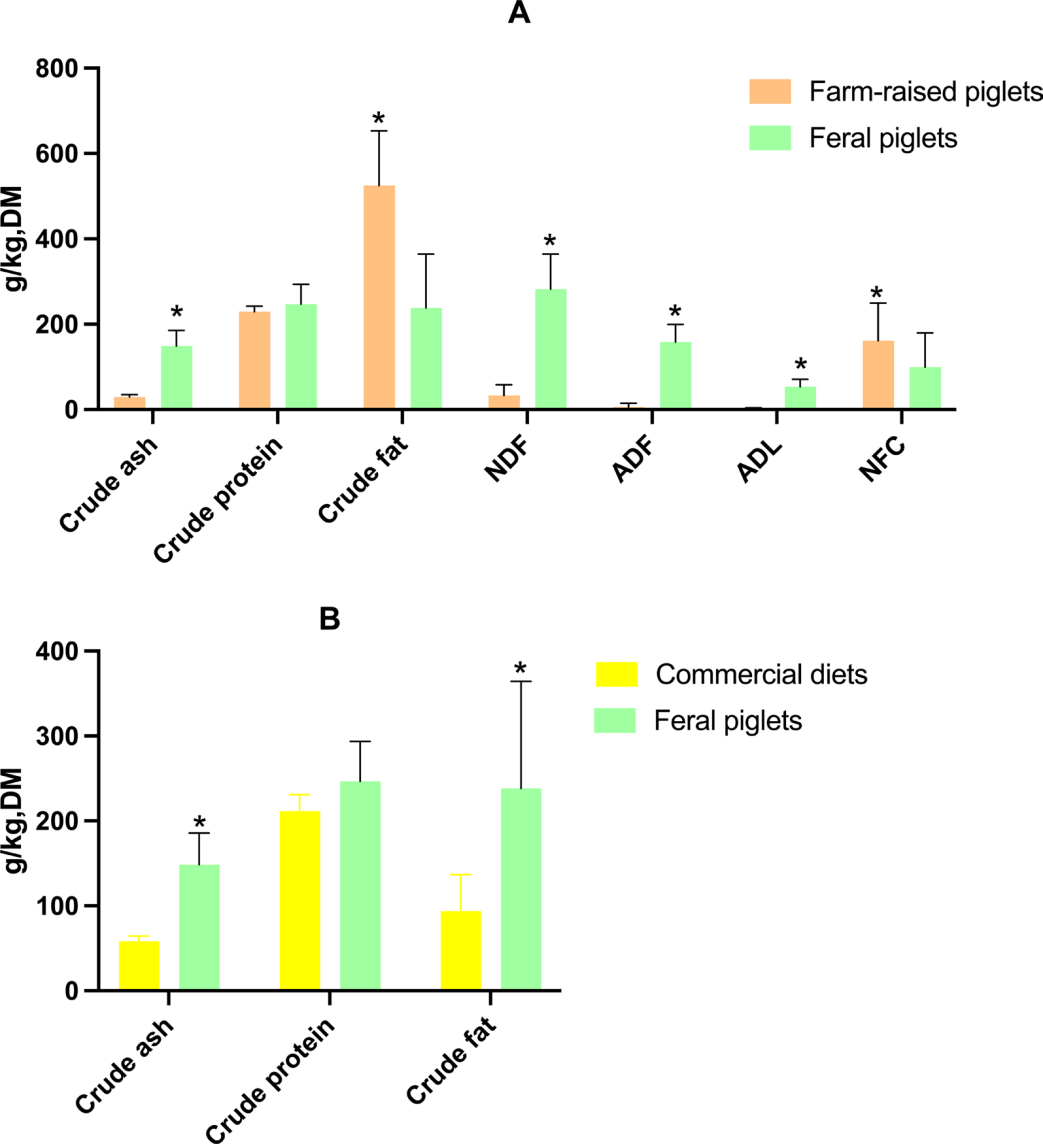
**A** The macronutrient profile of gastric content of farmed piglets in a Dutch farm (*n* = 14) and feral piglets (*n* = 13) living in Bahía Samborombón (Buenos Aires, Argentina) during the suckling phase; **B** The macronutrient profile of the commercial creep feeds and gastric content of feral piglets living in Bahía Samborombón (Buenos Aires, Argentina) Columns marked with * at the top indicate a significant greater value (*P* < 0.05). The error bars presented in the figure denote the standard deviation

**Fig. 2:**
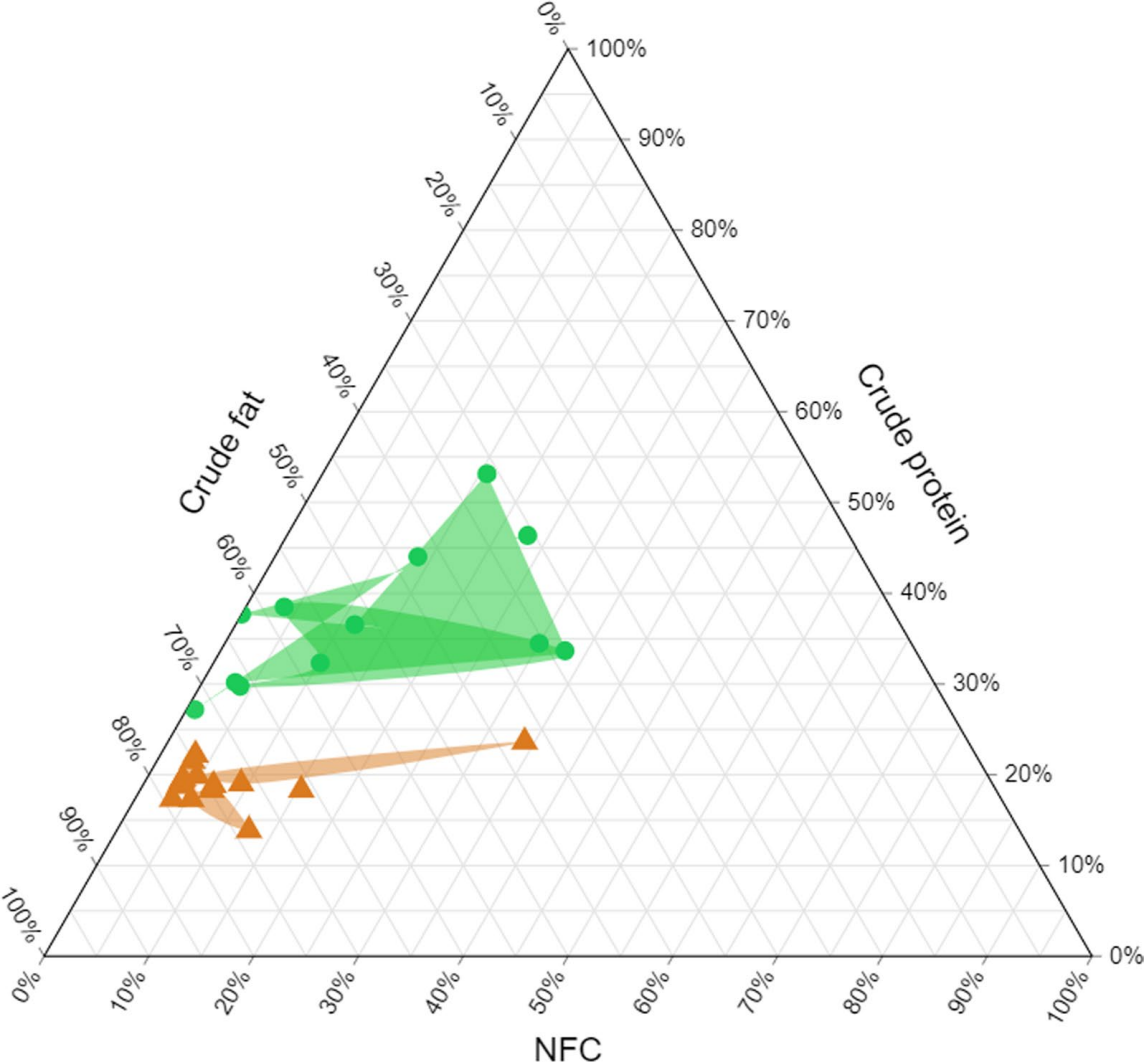
The ternary plot of crude protein, crude fibre, and NFC proportions on ME basis in gastric content of farm-raised piglets and feral piglets living in Bahía Samborombón (Buenos Aires, Argentina). Orange triangles: farm-raised piglets; green circles: feral piglets

As a result, each farmed piglet had an average colostrum intake of 466 g and an average creep feed intake of 620 g from day 14 of age until weaning. Higher dry matter values were observed in farmed piglet stomachs compared with feral piglet stomachs (233 vs. 148 g/kg, *P* < 0.05, data not shown in figures). In feral piglet gastric content, the crude ash proportion was three times greater than that in farmed piglet stomachs (Fig. 1A; 149 vs. 29 g/kg DM, *P* < 0.05). The crude protein concentration was similar between farmed and feral piglets (*P* > 0.05). The crude fat concentration was higher in farmed piglet stomachs compared to those of feral piglets (525 vs. 238 g/kg DM, *P* < 0.05). The NDF concentration on a dry matter basis in feral piglets was 282 g/kg, which was distinctly greater than the 36 g/kg observed in farmed piglets (*P* < 0.05). The ADF and ADL concentrations in gastric content of feral piglets were greater compared to farmed piglets (158 vs. 9 g/kg DM, 53 vs. 3 g/kg DM, respectively, *P* < 0.05). However, farmed piglets exhibited a higher proportion of NFC in their gastric content compared to feral piglets (161 vs. 99 g/kg DM, *P* < 0.05). Additionally, the calculated ME was also significant higher in the consumption of farmed piglets (2672 vs. 1499 kJ/kg DM, *P* < 0.05, data not shown in figures). On a ME basis (Fig. 2), no difference was seen in the energy derived from NFC. However, feral piglets showed a greater ME contribution from protein and a lower contribution from fat in their diet profile (*P* < 0.05).

**Fig. 3:**
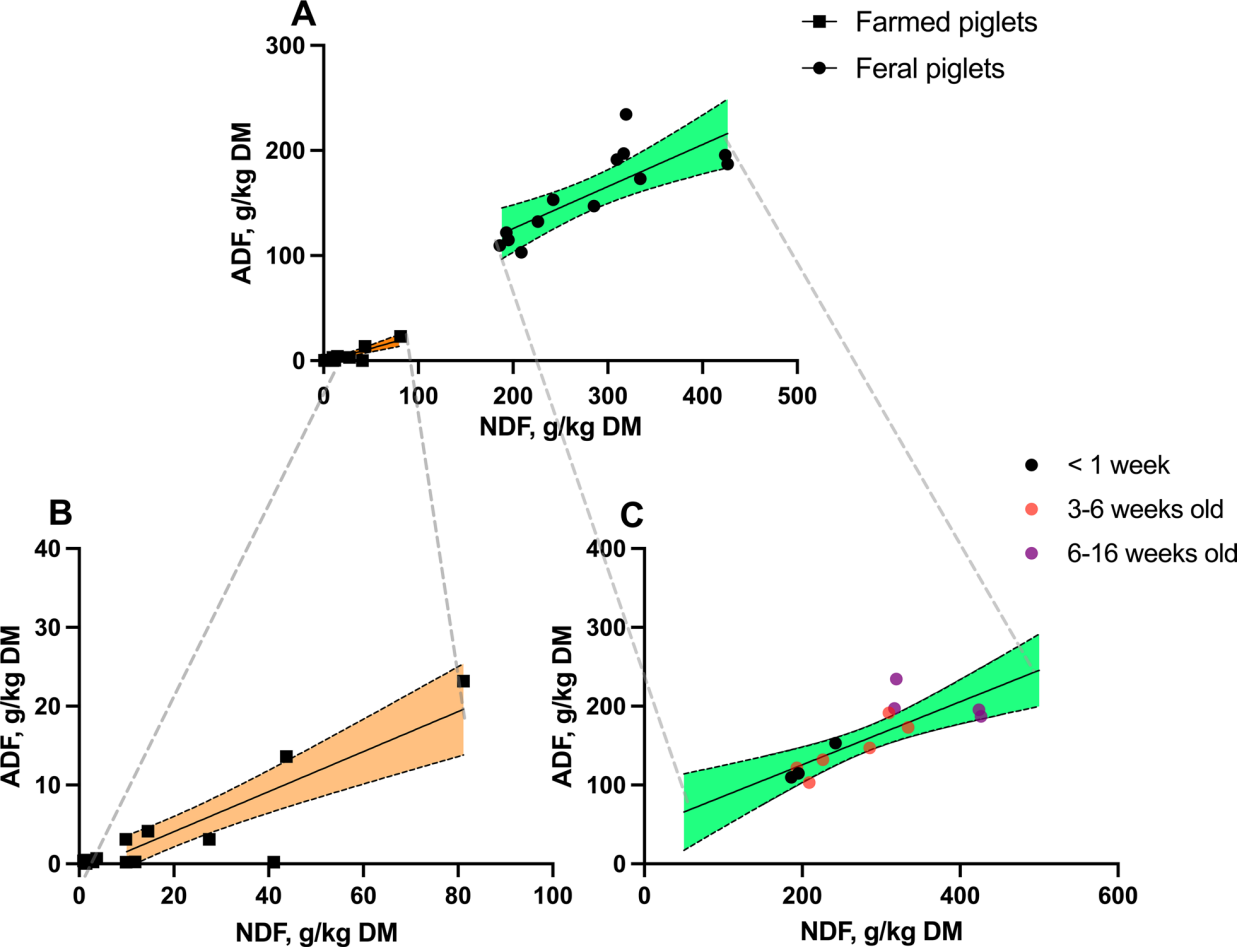
The linear regression curves between NDF and ADF levels in gastric content of farmed piglets living in a Dutch farm and feral piglets living in Bahía Samborombón (Buenos Aires, Argentina). **A** 95% confidence bands are shown in dotted lines and coloured areas. **B** Regression curve for farmed piglets only: Y = 0.2538 × X–0.9917 (*P* < 0.001, adjusted *R*^2^ = 0.77); **C** Regression curve for feral piglets only: Y = 0.3996 × X + 45.79 (*P* = 0.001, adjusted *R*^2^ = 0.64)

The correlation of NDF and ADF proportions were highly correlated in the stomach content of both feral piglets and farmed piglets (Fig. 3B, farmed piglets, R^2^ = 0.77, *P* < 0.001; Fig. 3C, feral piglets, R^2^ = 0.64, *P* = 0.001), with no significant difference in slopes (*P* = 0.78). However, combined regression curves in Fig. 3A still illustrated a considerable difference in ADF and NDF levels between feral and farmed animals. Meanwhile, a higher intercept was observed in the linear curve of feral piglets (*P* < 0.05), meaning that the ADF proportion within NDF was higher in feral piglet samples than in farmed piglet samples, irrespective of the NDF concentration as such.

**Fig. 4:**
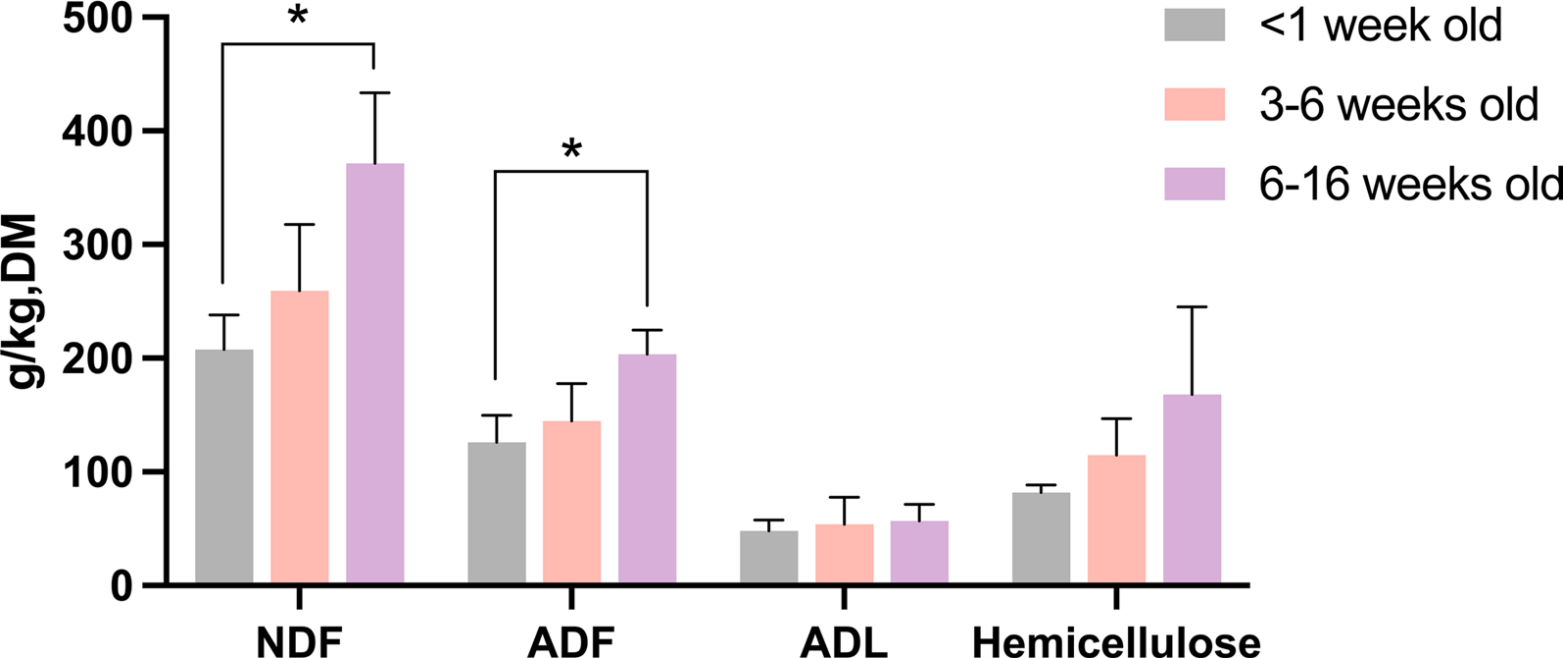
The comparison of fibre content in the gastric content of feral piglets from Bahía Samborombón (Buenos Aires, Argentina) across three age groups: < 1 week old, 3–6 weeks old and 6–16 weeks old

Notably, the feral piglets in 6–16 weeks old consumed significant higher proportion of NDF and ADF compared to those younger than 1 week old (Fig. 4, *P* = 0.007 and *P* = 0.009). Additionally, there was a trend of higher hemicellulose consumption of by 6–16 weeks old piglets compared to the youngest groups (*P* = 0.071).

### Comparison between gastric content of feral piglets and commercial creep feed

Unlike the variable gastric content, the nutrient contents of the commercial feeds were very similar (Fig. 1B). On a dry matter basis, the level of crude protein showed no difference between creep feed and feral piglet gastric content (*P* > 0.05). The ash concentrations were higher in gastric content (149 vs. 58 g/kg DM, *P* < 0.05). However, the ether extract concentration in commercial creep feed was lower than that in feral piglet stomachs (94 vs. 238 g/kg DM, *P* < 0.05).

## Discussion

In the wild, piglets’ dietary preferences are influenced by their surroundings, innate behaviour, and nutritional requirements for maintenance and moderate growth, rather than by pursuing maximal growth performance [14, 47]. In commercial farms, animals are typically fed for efficient growth performance, which has led to nutrient-dense and highly digestible diets [52]. However, the importance of early nutrition goes beyond covering actual nutrient requirements and affects performance and resilience throughout later life [19, 28, 40, 59]. The marked differences in gastric content nutrient profiles between feral and farmed suckling piglets, thus, raise the question whether the deviation from the natural diet in farmed conditions deserves more attention.

The high ash content in the stomachs of feral piglets is likely a result of rooting behaviour. The ingestion of underground plant parts with concomitant soil intake contributes to the intake of minerals such as calcium, iron and zinc, supporting the nutritional needs of pigs [2, 34]. Higher dietary iron intake likely, as highlighted in another study [25], suggests it may overcome the need for postnatal intramuscular iron injections. In addition, it has been demonstrated that soil and ash consumed by wild animals such as giant anteaters improve faecal consistency and may thus also modulate digestive kinetics [23]. The high ash content in feral piglet stomachs may thus add to the already high plant-based fibre content found in this study: NDF represents the plant cell walls including hemicellulose, cellulose and lignin. Together with the previously reported presence of leaves and stems in the stomach of these feral piglets [68], the finding of high NDF, ADF and ADL concentrations in the stomach content of feral piglets demonstrates the intake of coarse and fibrous matter at a young age — even before 1 week of age. The results indicating lower dry matter consumption of feral piglets, at 15% compared to 23% of farmed piglets, may also be attributed to the higher consumption of plant material by feral piglets in proportion to milk. Typically, the dry matter content of plant matter, such as leaves, fruits, grass and non-leaf tissues in the wild, ranges from 15 to 25% [56, 61]. In contrast, the dry matter of the feed applied in this study was 90%, and the dry matter of common commercial feed ingredients like corn and barley ranges from 85 to 90% [52]. However, whether natural preference or environmental availability primarily drives the eating behaviour of animals living in the wild is always a subtle point to determine. The hunting site in the current study, which features fresh water lagoons, grasslands, and islands with trees [51], may guide the diet choice of feral piglets. Studies on the diet pattern of feral piglets during the suckling phase in other continents are currently limited, but research on mature wild pigs in Europe, America, and Asia already reported that approximately 90% of the food resources consist of plant-origin materials [4, 9, 64]. We here demonstrate that this is happening already at a very young age before weaning. The higher ADF and ADL proportions within the NDF fraction in gastric content of feral piglets, along with the high correlation between NDF and ADF levels, suggests that feral suckling piglets ingest plant matter typically rich in insoluble fibres such as cellulose and lignin. Supplementation with insoluble fibre has been shown to promote gastrointestinal development, alleviate aggressive behaviour, and benefits the reproductive performance in growing pigs and sows [37, 38, 73]. Building on these findings, the addition of insoluble fibre in creep feed in farmed piglets was tested, resulting in the stimulation of intestinal development [67]. Moreover, given the gut-brain axis angle, higher gastric mobility and alteration of fermentation in the colon caused by insoluble fibre may further regulate the appetite signals of piglets at suckling phases [31, 36]. This suggests that exploring these “robust” ingredients as attractants holds promise for farmed piglets and may reduce the variability in feed intake of today’s commercial creep feeds.

The high ash and fibre content in the feral samples also complicates an adequate comparison of the other nutrients: as such, the crude protein concentrations were of similar magnitudes between commercial creep feeds and stomach contents of feral and farmed piglets, ranging from 21.2 to 24.7% on a DM basis. On an energy basis, protein is higher in feral piglet stomachs at the expense of fat. Creep feed intake is generally low compared with sow milk consumption [46, 50], whereas feral piglets already start eating plant material as early as the first week of age. Therefore, the difference in gastric fat and protein concentrations on an energy basis might arise from the difference in the proportion of solid feed intake. A higher proportion of easily digestible carbohydrates (NFC) was found in the stomach content of farmed piglets. However, the similar and relatively low energetic contribution from NFC in both breeds suggests that lactose from sow milk may have been the main origin in this case, especially given the high fat content in farmed piglet stomachs whereas creep feeds were distinctly lower in fat. The fat level in sow milk is around 60 g/kg on a DM basis, demonstrating that it constitutes the main fat resource in the gastric content of farmed piglets [11].

In (semi-)natural conditions, the suckling frequency is approximately 20% lower compared to domesticated herds living on farms, beginning from the second week of lactation [24, 33, 62]. Solid feed resources may thus contribute relatively more to the nutrient supply of these young piglets in the wild. Further studies are warranted to explore whether this high-fibre inclusion rate consumed by feral young piglets could potentially be leveraged to optimize nutritional interventions for farmed piglets.

## Conclusion

This study demonstrates distinct differences in the macronutrient profile of gastric content between feral and farmed suckling piglets, along with the disparity in commercial creep feed composition. Suckling feral piglets consume a diet with a high proportion of insoluble fibre and ash at a young age. The insoluble fibre intake increased progressively as feral piglets grew older. These nutrient shifts under farming conditions can be considered in the optimization of creep feed formulation to tackle lingering challenges associated with abrupt weaning in farms.

## Data Availability

The data that support the findings of this study are available from the corresponding author upon reasonable request.
